# The effect of oxidant species on direct, non-syngas conversion of methane to methanol over an FePO_4_ catalyst material†

**DOI:** 10.1039/c9ra02327e

**Published:** 2019-09-30

**Authors:** Venkata D. B. C. Dasireddy, Darko Hanzel, Krish Bharuth-Ram, Blaž Likozar

**Affiliations:** Department of Catalysis and Chemical Reaction Engineering, National Institute of Chemistry Hajdrihova 19 1001 Ljubljana Slovenia dasireddy@ki.si +386 1 4760300 +386 1 4760504; Department of Low and Medium Energy Physics, “Jozef Stefan” Institute Jamova cesta 39 1000 Ljubljana Slovenia; Physics Department, Durban University of Technology Durban 4000 South Africa; School of Chemistry and Physcis, University of KwaZulu Natal Durban 4000 South Africa

## Abstract

The effect of the phase transformation of a FePO_4_ catalyst material from the tridymite-like (tdm) FePO_4_ to the α-domain (α-Fe_3_(P_2_O_7_)_2_) during the direct selective oxidation of methane to methanol was studied using oxidant species O_2_, H_2_O and N_2_O. The main reaction products were CH_3_OH, carbon dioxide and carbon monoxide, whereas formaldehyde was produced in rather minute amounts. Results showed that the single-step non-syngas activation of CH_4_ to oxygenate(s) on a solid FePO_4_ phase-specific catalyst was influenced by the nature of the oxidizer used for the CH_4_ turnover. Fresh and activated FePO_4_ powder samples and their modified physicochemical surface and bulk properties, which affected the conversion and selectivity in the partial oxidation (POX) mechanism of CH_4_, were investigated. Temperature-programmed re-oxidation (TPRO) profiles indicated that the type of moieties utilised in the procedures, determined the re-oxidizing pathway of the reduced multiphase FePO_4_ system. Mössbauer spectroscopy measurements along with X-ray diffraction (XRD) examination of neat, hydrogenated and spent catalytic compounds, demonstrated a variation of the phosphate into a mixture of crystallites, which depended on operating process conditions (for example time-on-stream). The Mössbauer spectra revealed the change of the initial ferric orthophosphate, FePO_4_ (tdm), to the divalent metal form, iron(ii) pyrophosphate (Fe_2_P_2_O_7_); thereafter, reactivity was governed by the interaction (strength) with individual oxidizing agents. The Fe^3+^ ↔ Fe^2+^ chemical redox cycle can play a key mechanistic role in tailored multistep design, while the advantage of iron-based heterogeneous catalysis primarily lies in being inexpensive and comprising non-critical raw resources. When compared to the other catalysts reported in the literature, the FePO_4_-tdm phase catalysts showed in this work exhibited a high activity towards methanol *i.e.*, 12.3 × 10^−3^ μmol_MeOH_ g_cat_ h^−1^ using N_2_O as an oxidant. This catalyst also showed a high activity with O_2_ as an oxidant (5.3 × 10^−3^ μmol_MeOH_ g_cat_ h^−1^). Further investigations will include continuous reactor unit engineering optimisation.

## Abstract 2

The effect of the phase transformation of a FePO_4_ catalyst material from the tridymite-like (tdm) FePO_4_ to the α-domain (α-Fe_3_(P_2_O_7_)_2_) during the direct selective oxidation of methane to methanol was studied using oxidant species O_2_, H_2_O and N_2_O.

## Graphical abstract

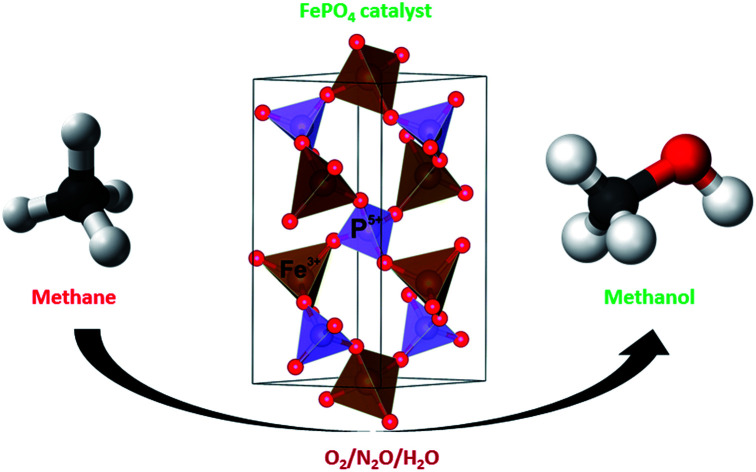

## 1 Introduction

The direct conversion of methane to methanol has been attracting considerable attention because of its great potential application in the efficient utilization of abundant natural gas reserves. In the last decade a number of interesting approaches was suggested for the effective implementation of this difficult transformation.^1^ A most attractive approach is to convert the natural gas into products such as methanol, which under ambient temperature and pressure is a liquid.^2^ However, over the past few decades, the conversion of methane to methanol has remained as one of the major unrequited challenges in chemistry. To activate methane, usually high temperatures are required. At these temperatures, formed methanol undergoes further oxidation to CO_2_ and H_2_O, as illustrated below.^3,4^1
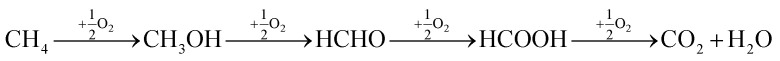


Methanol is formed *via* methane oxidation by α-oxygen, CH_4_ + (M^n^–O*^−^)_α_, migrated from α-oxygen sites. It is generally accepted that α-sites perform the oxidation *via* the reversible redox transition M^*n*^ ↔ M^*n*+1.3^. Much of the work on the variety of catalysts that have been investigated for partial oxidation of methane to methanol has been summarized in recent literature reports.^5–7^ The majority of the studies involved supported metal oxide catalysts, primarily vanadium and molybdenum oxide.^1,2^ Higher activity and selectivity to the desired products over mixed metal oxide catalysts can be attributed to the formation of easily reducible metal oxide species caused by interactions between the metals. Loading of supported phase below the monolayer coverage has been shown to be preferable for the high production of formaldehyde and methanol from methane.^8,9^ Otsuka *et al.*^1,10^ reported that the conversion of methane is accelerated by co-feeding hydrogen with oxygen over several iron containing catalysts. The co-feeding of hydrogen induces the formation of methanol over FePO_4_, FeAsO_4_ and FAPO-5 (Fe : Al : P = 0.1 : 0.9 : 1.0) catalysts at atmospheric pressure and in the temperature range of 350–500 °C. Thus, in order to design a better catalyst, it is quite important to understand which the effective and/or ineffective iron sites are in the selective oxidation of methane to methanol.

A low temperature (150 °C), isothermal, gas-phase recyclable process was described for the partial oxidation of methane to methanol over Cu/ZSM-5 by Sheppard *et al.*,^11^ which showed a stable formation of methanol for a long period of time. Depending on the iron content and activation conditions, a variety of Fe species may be available in the zeolite, ranging from isolated Fe(ii) and Fe(iii) cations and oligo-nuclear Fe complexes up to large agglomerates of iron oxide. FeZSM-5 zeolites have a long application history as catalysts for oxidations by N_2_O.^3,4,12^ Methanol and dimethyl ether (DME) were the products extracted from the catalytic surface. Co-feeding water strongly increased methanol selectivity, which attained a fractional concentration of 62% at 275 °C. The location, dispersion and environment (acidic or alkaline) of iron sites and the nature of oxidant are key factors in determining the catalytic performances of iron-containing mesoporous materials for selective oxidation reactions.^13,14^ Shiota and Yoshizawa^15^ have computed and analysed the reactions of the first row MO^+^ complexes (M = Sc, Ti, V, Cr, Mn, Fe, Co, Ni and Cu) and methane, which can competitively form methanol and methyl radical. Anderson *et al.*^16^ carried out a systematic study of the conversion of methane using a number of metal oxide catalysts. They demonstrated that cobalt oxide is the most active single component catalyst which resulted in a high conversion, but with a very low selectivity towards methanol synthesis.

Štolcová and co-workers^17,18^ examined the influence of structure and reactivity of copper iron pyrophosphate catalysts for the selective oxidation of methane using O_2_ and N_2_O as oxidizing agents. These oxidants showed appreciable impact on the onset of both methane conversion and the primary oxidation products. The catalytic results showed that the lattice oxygen of the catalyst could react with methane molecules producing methanol and that replenishment of the lattice oxygen by N_2_O takes places rather readily and rapidly. Wang *et al.*^13^ showed the use of N_2_O oxidant for the epoxidation of C_3_H_6_ over iron-containing catalysts. Iron is peculiar for obtaining high selectivity to propylene oxide, and the modification of the iron sites with an alkali metal salt can promote the C_3_H_6_ epoxidation. Christos *et al.*^19^ have shown that the reactivity of commercial zeolite-based catalysts containing Fe and/or Cu cations for the partial oxidation of methane is influenced by the acid sites strength and concentration in the catalyst which depends on the Si/Al molar ratio and type of zeolite. It was shown that the Fe cations are responsible for the superior oxygenates productivity, while the crucial role of Cu is to maintain high MeOH selectivity by suppressing the production of the deeper oxidation product, HCOOH. Partial oxidation of methane over iron phosphate supported on silica produced high formaldehyde yields.^17,20^ In the literature,^18,21^ it has been shown that the nature of the oxidant, however, was observed to play a vital role in favoring yield towards methanol. In line with the state of the art direct conversion of methane to methanol, we have undertaken this work with the aim to investigate which of the oxidant species, O_2_, H_2_O or N_2_O, would lead to achieving a high yield of methanol over FePO_4_ catalysts. As an attempt to achieve this objective, an ammonia gel method was employed to synthesize FePO_4_ catalyst, the resulting catalyst structures were then revealed by different characterisation techniques and the influence of oxidants on the structure and phase of FePO_4_ during the reaction was examined.

## 2. Experiment

### 2.1 Synthesis of the catalyst

The catalysts were synthesized by the ammonia gel method described by Friedrich *et al.*^22–24^ This catalytic preparation methodology was chosen because of its simplicity and to achieve a better control of particle size and morphology of the active phase. In essence, an appropriate amount of ferric nitrate, Fe(NO_3_)_3_·9H_2_O (99%, Sigma Aldrich) was dissolved in water and then a dilute ammonium solution (25% NH_3_ in H_2_O, Sigma Aldrich) was added. This led to precipitation and formation of iron(iii) hydroxide (brown gel). Orthophosphoric acid (85% H_3_PO_4_, Sigma Aldrich) was added while stirring the gel, followed by a 40 wt% silica solution. The stirred mixture was then heated to 60 °C and kept at this temperature for 2 h. The obtained gel was heated at 90 °C for 12 h, and the dried solid achieved was then calcined at a temperature 500 °C for 4 h in a 2 bar flow of air.

### 2.2 Characterization of catalysts

The characterizations of fresh and used catalysts are described below. Nitrogen physisorption analyses were carried out by degassing the catalysts under N_2_ flow for 4 h at 200 °C. The degassed samples were analysed in the Micromeritics ASAP 2020 multi-point surface area and porosity analyser. Powder X-ray diffraction (XRD) studies were conducted using the PANalytical X'Pert Pro instrument. Scanning from 10 to 90° was carried out using the CuKα-radiation source with the wavelength of 0.15406 nm. Temperature programmed reduction-oxidation (TPRO) was performed using the Micromeritics 2920 Autochem II Chemisorption Analyser. Initially, the reduction of the catalyst was done using 4.9 mol% H_2_ in Ar as a reducing agent as in the method described in.^25,26^ Temperature programmed desorption (TPD) was carried out using the Micromeritics 2920 Autochem II Chemisorption Analyser as well. After reduction, the catalysts were pre-treated at 350 °C under the stream of helium for 60 min. The temperature was consequently decreased to 80 °C. Appropriate pre-chosen gas was passed over the catalysts (10 mol% CO_2_ in He or 4.9 mol% H_2_ in Ar) at the flow rate of 30 mL min^−1^ for 60 min. The excess gas was removed by purging with helium for 30 min. The temperature was thereafter gradually raised to 900 °C by ramping at 10 °C min^−1^ under the flow of helium, wherein the desorption data of CO_2_ or H_2_ was recorded. The desorption data of O_2_, H_2_O and N_2_O were also recorded in the same procedure. Metal dispersion is calculated using CO chemisorption which is illustrated in literature^24,26^

The structural morphology of the prepared catalysts was studied using field-emission scanning electron microscope (Carl Zeiss, FE-SEM SUPRA 35VP), equipped with energy-dispersive X-ray spectroscopy hardware (Oxford Instruments, model INCA 400). Particle size, morphology and elemental mapping performed by EDXS analyses were investigated using Cs corrected scanning transmission electron microscope JEOL ARM 200 CF equipped with JEOL Centurio 100 mm^2^ EDXS system. ^57^Fe-Mössbauer spectroscopy measurements were made at room temperature (RT) in conventional transmission geometry with a ^57^Co source embedded in Rh matrix.

### 2.3 Partial oxidation of methane

The catalytic partial oxidation runs using the FePO_4_ catalysts were carried out in a horizontal fixed-bed U-shaped quartz reactor as described in.^27,28^ The catalyst (∼0.4 g) was placed in the middle of the reactor and a flow of N_2_ (30 mL min^−1^) was introduced into the reactor at a temperature of 200 °C in order to remove the physisorbed gases from the surface of the catalyst. The catalytic runs were carried out under atmospheric pressure at the temperature range of 200–500 °C using undiluted high purity CH_4_ (99.95%) and the appropriate oxidant (O_2_, N_2_O or H_2_O) at flow rate of 60 mL min^−1^, corresponding to the gas hour space velocity (GHSV) of 3600 h^−1^ with a methane to oxidant ratio of 1 : 1. The gas products were analysed using an Agilent 490 Micro GC TCD equipped with CP-Molsieve and PoraPolt U columns. Reported values are given after 5 h of the reaction under steady-state conditions. The product mixture including methane, methanol, carbon oxides and nitrogen were analysed by online quadrupole mass spectrometry (MS). The signals in the MS are calibrated with different mole fractions of methane, methanol, carbon oxides and nitrogen in order to determine the mole composition of gases in the outflow. No other gaseous products were detected during the reaction. All the data points were recorded in duplicate with a standard deviation of ± 2%. The carbon mass balances are in the range of 98–99%.

The methane conversion (*δ*) was calculated using the following equation:
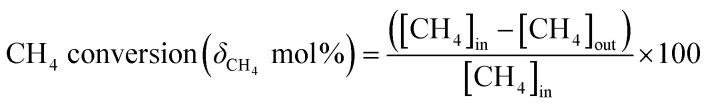

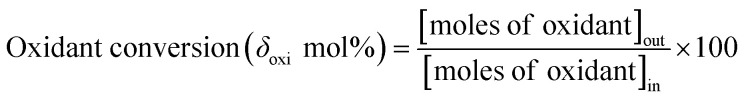

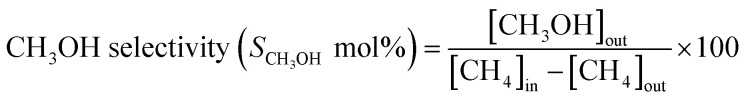


## 3 Results and discussion

**Fig. 1:**
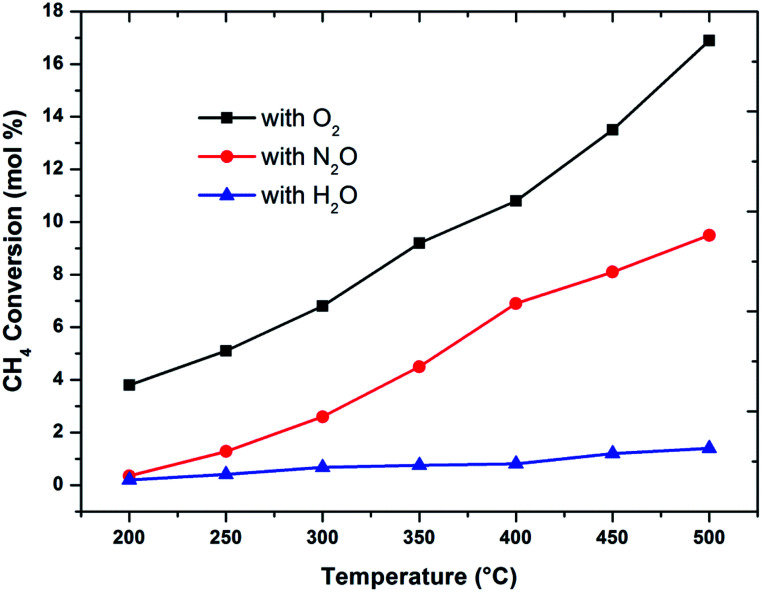
Influence of oxidant on methane conversion over FePO_4_ catalyst with varying temperature (GHSV = 3600 h^−1^ and methane to oxidant ratio of 1 : 1).

The effect of reaction temperature on methane conversion over FePO_4_ catalyst with different oxidative environments was investigated as shown in Fig. 1. Methane conversion with N_2_O and O_2_ increases with temperature; however when H_2_O is used as an oxidant, methane conversion increases at much lower linear rate with temperature. Furthermore, the maximum methane conversion (17%) was obtained when oxygen is used as an oxidant at a temperature of 500 °C, the highest reaction temperature in this study. The differences in conversion rates clearly show that selective oxidation of methane on FePO_4_ is influenced by the nature of the oxidant. These facts strongly suggest that the reaction mechanisms are different.^29^ This conclusion is strengthened by the greater conversions with O_2_ than N_2_O, which is in agreement with the reaction thermodynamics and the activation energy differences between these two oxidants.^1^ This means that there exists a lower kinetic barrier for O_2_ than N_2_O in catalytic selective oxidation of methane.

**Fig. 2:**
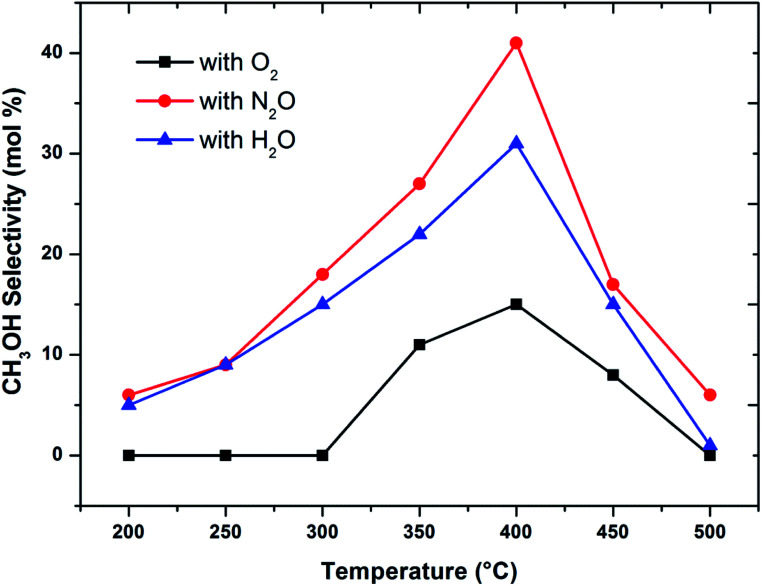
Influence of oxidant on CH_3_OH selectivity in methane partial oxidation reaction over FePO_4_ catalyst with varying temperature (GHSV = 3600 h^−1^ and methane to oxidant ratio of 1 : 1).

When considering the effect of temperature on the methane conversion, two factors need to be considered. Firstly, methane conversion will depend on the oxidant feed concentration, secondly, even if total oxidant consumption occurs, methane conversion can change with a change in selectivity. Most of the studies^2,3,11,18,30,31^ reported in literature have examined the effect of temperature over a range varying from approximately 300 to 500 °C. It has been found that very low conversion occurs until a critical temperature is reached, after which a very rapid rise in conversion is observed (Fig. 1). This usually corresponds to total oxidant consumption. Typically the products obtained in the partial oxidation of reaction consist of CH_3_OH, CO, CO_2_, HCHO and H_2_O. CO_2_ and H_2_O are formed initially at temperatures below 300 °C. At 400 °C both selectivity and yield of methanol are found to pass through a maximum before decreasing as the temperature is increased further (Fig. 2). Methanol formation is accompanied by the production of CO along with CO_2_ and H_2_O. It was generally found that increasing the temperature well above the self-ignition temperature of methanol favors the production of CO, CO_2_ and H_2_O at the expense of methanol.^1,10,18^

**Fig. 3:**
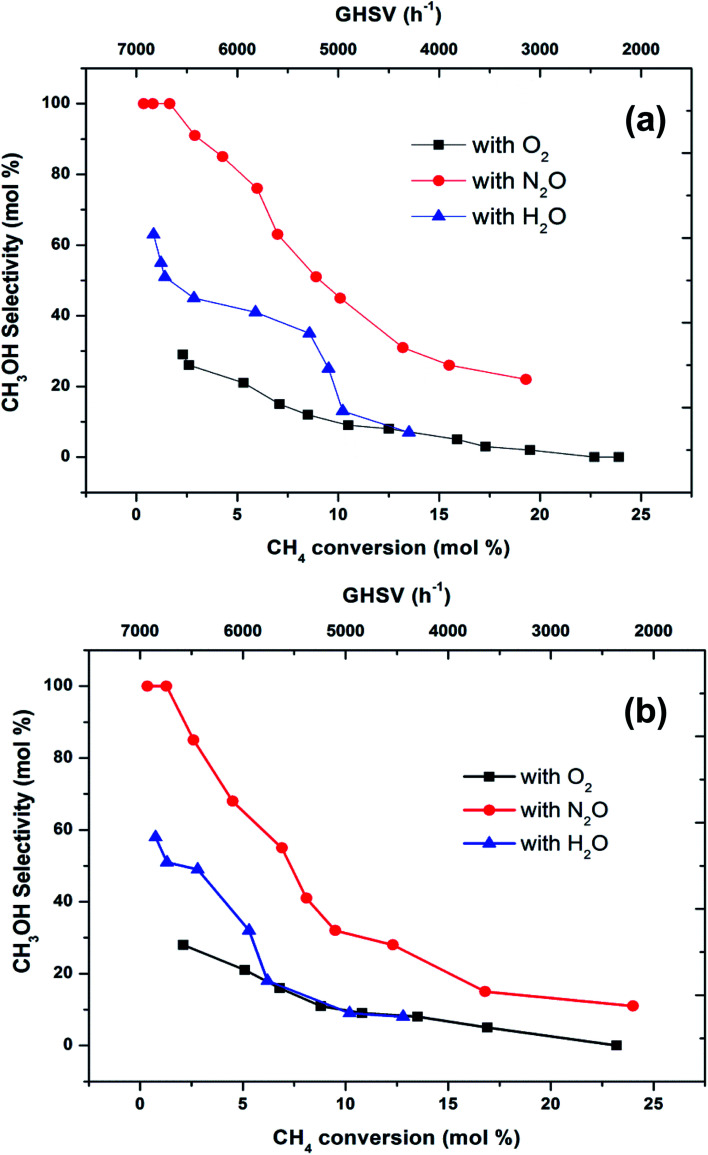
Selectivity towards methanol varying with methane conversion over FePO_4_ catalyst at the temperatures of (a) 300 °C and (b) 400 °C (GHSV = 2000–7000 h^−1^, methane to oxidant ratio of 1 : 1).

To make a better comparison of catalytic performance among the oxidants, we further carried out the reactions with various flow rates between a GHSV of 2000 and 7000 h^−1^. The conversion of methane increased and the selectivity to methanol decreased with decrease in the flow rate as expected (ESI, Fig. S1†). From these data, the plot (Fig. 3) of methanol selectivity *versus* methane conversion was constructed. The selectivity towards methanol was very high at lower methane conversion at higher flow rate. However, when the comparison was made at higher methane conversion, the selectivity to methanol was the highest when N_2_O is used as oxidant. In addition to that, the selectivity of methanol at low conversions (<2%) of methane was almost identical (see first two data points at nearly 100% selectivity in Fig. 3). By elevating the reaction temperature at these conditions also, methanol selectivity gradually dropped and raised the CO production. This was a consequence of the low stability of methanol at higher temperatures and thereby it over oxidised to carbon oxides. It has been well recognized that the selective oxidation of methane proceeds *via* redox mechanism, but the pathways and product distribution depend on the nature of the oxidant and the reaction conditions.^16,29^

**Fig. 4:**
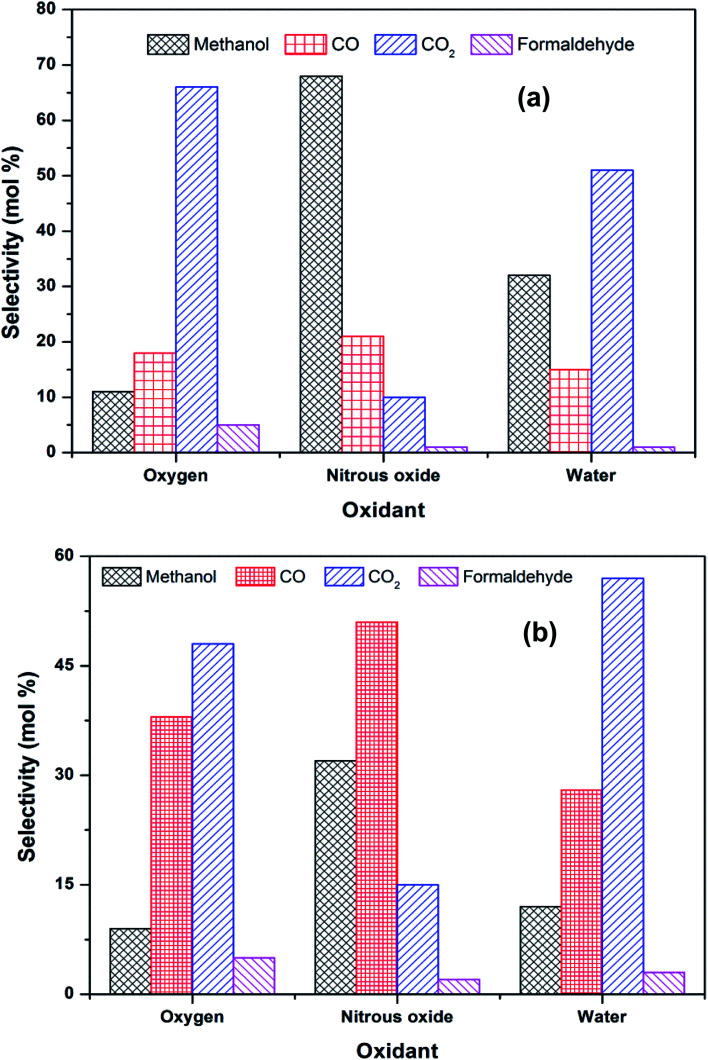
Selectivity towards products in the partial oxidation of methane at an iso-conversion of (a) 5% and (b) 10% over FePO_4_ catalyst with various oxidants (temperatures of 400 °C and methane to oxidant ratio of 1 : 1).

Activation of methane may occur by both hemolytic and heterolytic mechanisms.^2,27^ Thus the investigations of used catalysts of FePO_4_ might give an insight into the modified physicochemical properties of the catalysts which further influenced the conversion and selectivity in the partial oxidation of methane. The rate determining step would be the rupture of the C–H bond and the formation of CH_3_ and HO radicals were postulated as the initial step.^1,29^ For methane oxidation to methanol, the most strongly supported mechanism consists of consecutive conversion scheme as shown in eqn (1). The methoxy radical is an important intermediate in the reaction pathway.^19^ Elimination of hydrogen from methoxy radical in a reaction such as oxidative dehydrogenation gives formaldehyde which is easily converted to CO and CO_2_. Hydrogenation of the methoxy radical yields methanol.^1^ Stabilization of the methoxy radical by hydrogenation is a key step to achieve a high methanol yield.^18^Fig. 4 shows the selectivity towards products in the partial oxidation of methane at iso-conversions of 10% and 5%. At both iso-conversion conditions (similar conversions at the same temperature), methanol was formed in high quantity when N_2_O used as an oxidant. CO_2_ is the major product with both O_2_ and H_2_O. This could be either due to the further oxidation of methoxy radical to CO_2_ or the direct combustion of methane to CO_2_. On the other hand, when N_2_O was used, these effects were less systematic; the production rate of methanol was approximately doubled (Fig. 4). Using N_2_O, methanol selectivity was higher, with decreased CO_2_ selectivity. The catalytic differences have been ascribed to the differing oxidizing power of O_2_, N_2_O and H_2_O. In accordance with the Mars–van Krevelen mechanism,^1,2,29^ which has been proposed, the re-oxidation of the catalyst will be less effective with N_2_O and H_2_O compared to O_2_. At all conditions, formaldehyde is formed in very minor quantities, which could also be due to a secondary oxidation of formaldehyde to CO or CO_2_.

**Fig. 5:**
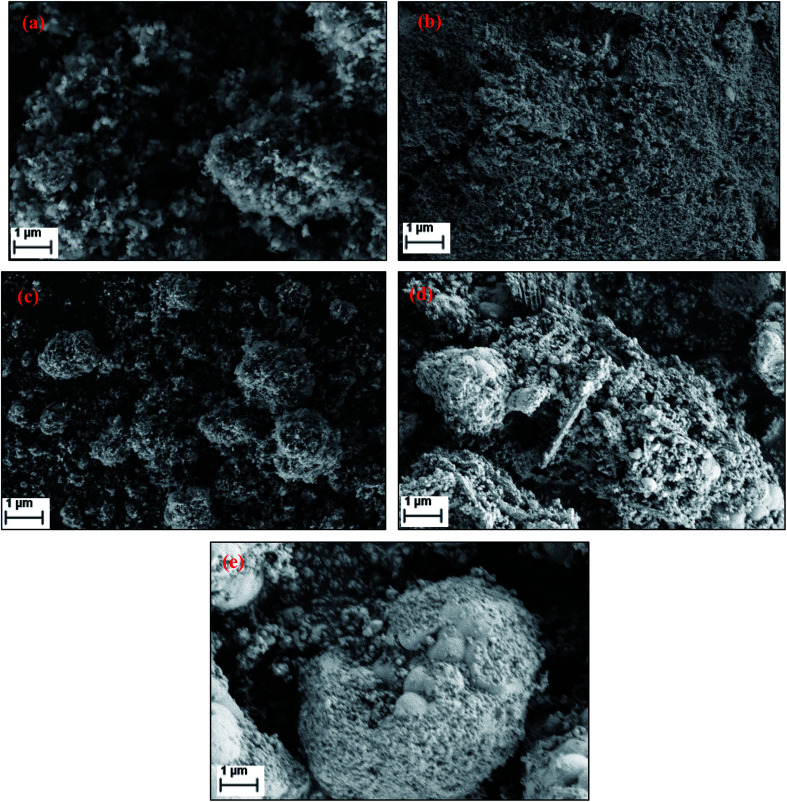
Scanning electron micrographs (SEM) of FePO_4_ catalysts under various conditions (a) fresh, (b) reduced under CH_4_, (c) oxidized with O_2_, (d) oxidized with N_2_O and (e) oxidized with H_2_O.

Fig. 5 shows the SEM images of FePO_4_ catalysts treated under various conditions. The fresh catalyst possesses rough crystalline morphology with a particle sizes ranging from 50–80 nm with a homogeneous dispersion of the particles. After reduction, the agglomeration of these particles was observed. After oxidation with oxygen the crystalline nature is retained with a separate agglomerated bulk particles. After oxidation with N_2_O and H_2_O, the catalysts showed a relatively poor crystalline structure and an amorphous like morphology (Fig. 5d and e). The BET surface areas, measured by the physical nitrogen adsorption for all of the samples, are presented in Table 1. The specific surface area of fresh FePO_4_ was found to be 19 m^2^ g^−1^. However, BET surface area decreased after reduction and oxidation treatments. After reduction, the surface area of the catalyst reduced drastically to 9 m^2^ g^−1^, due to the blocking of the pores of the FePO_4_ by the amorphous carbon which formed large crystallites, as evidenced by XRD and pore-size distribution measurements.

**Table 1:** Particulate properties of fresh, reduced and oxidized FePO_4_ nanocomposite catalysts

Catalyst condition	Surface area (m^2^ g^−1^)	Pore volume (cm^3^ g^−1^)	Metal dispersion^a^ (%)	Crystallite size^b^ (nm)
Fresh	19	0.021	27.8	28
Reduced (with CH_4_) catalyst	9	0.015	10.3	41
Oxidized (with O_2_) catalyst	15	0.020	22.3	32
Oxidized (with N_2_O) catalyst	12	0.019	17.3	35
Oxidized (with H_2_O) catalyst	13	0.018	15.8	35

a Calculated from CO chemisorption^32^.

b Average crystallite size calculated by Scherrer equation.

The N_2_ adsorption–desorption isotherms of the fresh, reduced and oxidised catalysts (ESI, Fig. S2†) can be categorised as the type IV isotherms, with a distinct hysteresis loop, observed in the relative pressure (*P*/*P*_o_) range of 0.47–0.79.^33^ The pore-size distribution, calculated from the desorption counterpart using Barrett–Joyner–Halenda (BJH) method, showed a dominant peak in the mesoporous range (ESI, Fig. S3†). The metal dispersion of Fe species was calculated from CO chemisorption. Metal dispersion showed the similar trend to surface area, as the fresh catalyst showed a high metal dispersion compared to the reduced and oxidised catalysts. Among the oxidised catalysts, the catalyst oxidised with oxygen showed a higher metal dispersion compared to the catalysts oxidised with N_2_O or H_2_O. This could be due to the amount of available oxygen present in N_2_O or H_2_O. This shows that the nitrous oxide or water provides adequate oxidation of reduced sites of the catalyst. This may explain the fact that the number of active sites is altered and the nature of the active sites of the oxidised catalyst remained unchanged.

**Fig. 6:**
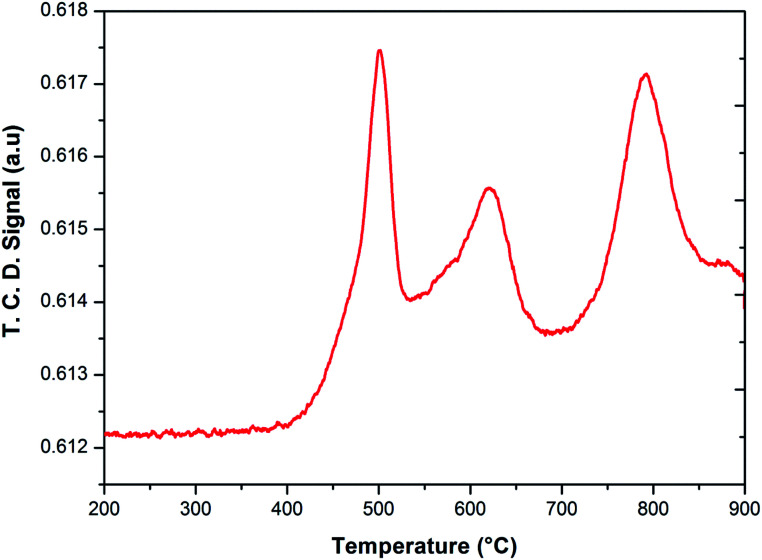
Temperature programmed reduction of FePO_4_ catalyst under 10% CH_4_ in Ar.

To investigate the phase transformations occurred during reduction and oxidation, powder XRD and Mössbauer analysis of the reduced and oxidized samples were carried out. The reduced sample was obtained using reducing the fresh FePO_4_ catalyst under 10% CH_4_ in Ar. In the TPR profile, the catalyst showed three peaks at the temperatures of 492, 625 and 815 °C (Fig. 6). These peaks represent reduction of FePO_4_ to Fe_2_P_2_O_7_ as suggested in:^22,34^
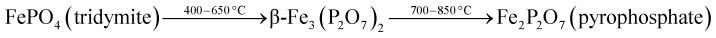


As reported in the literature,^35,36^ the first step in the reduction occurs above the temperatures of 500 °C. The use of CH_4_ as reductant in this study accelerated the reduction, probably due to the activation and spill over of hydrogen from the metal centers to the iron phosphate.

**Fig. 7:**
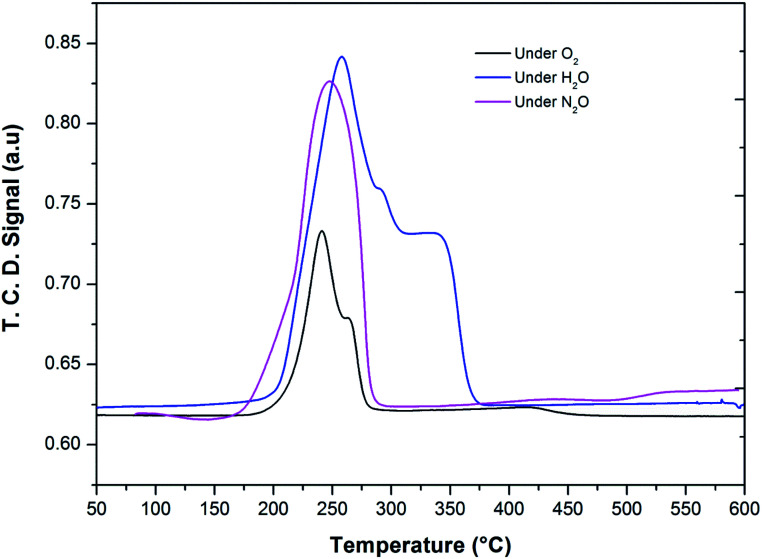
Temperature programmed reduction–oxidation (TPRO) profiles of FePO_4_ catalyst under various oxidant environments.

**Fig. 8:**
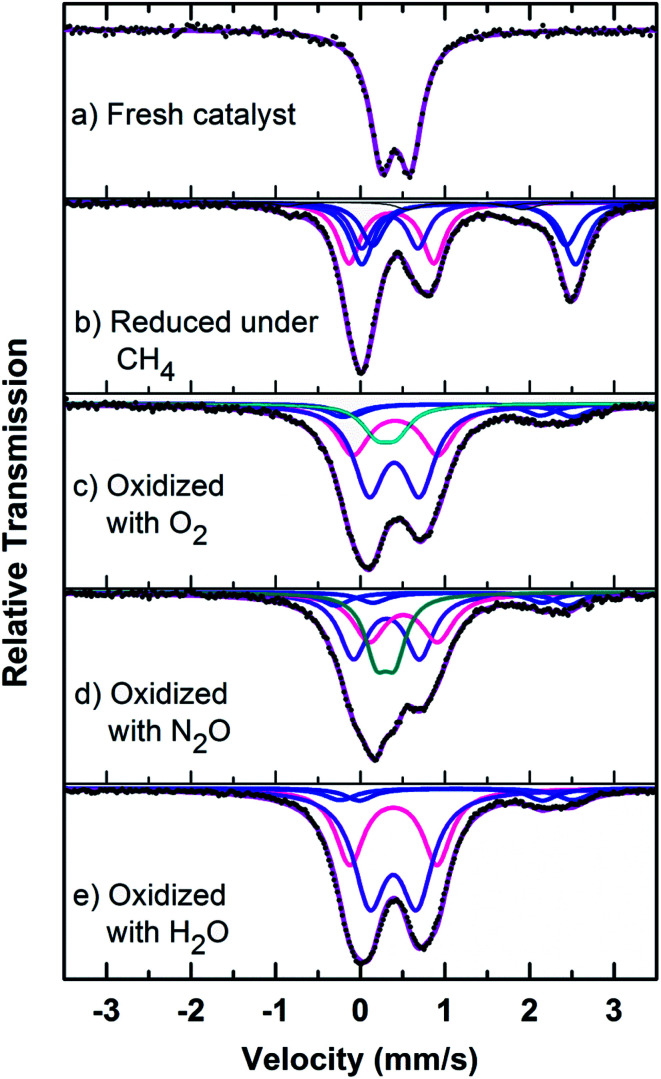
Mössbauer spectra of fresh, reduced (with CH_4_) and oxidized (with N_2_O, O_2_ and H_2_O) FePO_4_ catalyst.

After the reduction, the oxidation was conducted using O_2_, N_2_O and H_2_O as oxidants (10% of oxidant in Ar), separately. The TPRO profile under H_2_O exhibited a very wide range of oxidation profile in the temperature ranging from 200–370 °C (Fig. 7). When N_2_O is used an oxidant, only one peak was exhibited and two peaks in the profile occurred with O_2_ as oxidant. There were no peaks observed above 500 °C, in the TPRO profiles. Various literature reports^37,38^ stated that the oxidative transformation of iron pyrophosphate phase to quartz phase occurs in two steps as below:
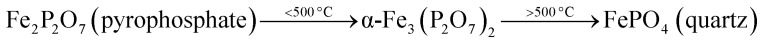


The formation of the quartz type phase has been reported in the literature; however, it was formed at temperatures above 500 °C.^39^ However, it was also observed that the transformation between the α-phase and Fe_2_P_2_O_7_ is reversible. Thus, from TPRO profiles, it is evident that the type of oxidant used in the re-oxidation influenced the re-oxidation path way of reduced FePO_4_ catalyst. The powder XRD patterns of the fresh, reduced and oxidized catalysts are shown in Fig. 8.

The fresh catalyst showed the presence of the FePO_4_ trydimite-like (tdm) phase by exhibiting a main peak at a 2*θ* = 34° and minor peaks in the range of 24–30°. The XRD pattern of the reduced catalyst (with methane) shows two distinct peaks at 2*θ* values of 24° and 30°, confirming the formation of the Fe_2_P_2_O_7_ phase range (ESI, Fig. S4†). The formation of this phase is also observed in literature^22,37^ when FePO_4_ is reduced under various hydrocarbon reduction atmospheres. When the reduced catalyst is oxidised in the presence of oxygen, the formation of the α-phase (α-Fe_3_(P_2_O_7_)_2_) is observed. The XRD profile of this catalyst showed two characteristic major peaks in the region of 2*θ* = 33–36° indicating the presence of α-phase (α-Fe_3_(P_2_O_7_)_2_) along with some minor peaks. Some of the XRD peaks in this region also coincide with peaks characteristic of the β-phase.^22,37^ The XRD profile of the catalyst oxidized with N_2_O also showed the presence of the α-phase along with Fe_2_P_2_O_7_ phase, but absence of the β-phase which would give a peak at 2*θ* = 36° range (ESI, Fig. S4†). Oxidation of the catalyst in H_2_O showed a similar pattern to the catalyst oxidized with oxygen, but with the notable absence of a peak at a 2*θ* = 28°, indicating the absence of the β-phase under H_2_O oxidation also. This could be due to a high oxidizing atmosphere being required for the formation of β-phase and the low oxidizing strength of H_2_O compared to O_2_.^22,37^

Mössbauer spectra of fresh, reduced (with CH_4_) and oxidized (with N_2_O, O_2_ and H_2_O) FePO_4_ catalysts are shown in Fig. 8. The spectra were corrected for thickness effects and then fitted with the analysis code RECOIL^51^ using Lorentzian line shapes for the spectral components. The spectral fit parameters (isomer shift (IS), electric quadrupole splitting (QS), line width (HWHM), area fractions (*f*)) and phase assignments are collected in Table 2 where the isomer shifts are given relative to α-Fe at room temperature. The phase assignments were made on the basis of parameters reported in ref. 36, 37 and 40–42.

**Table 2:** Mössbauer parameters, isomer shift (IS), electric quadrupole splitting (QS), *Γ* (HWHM), and the attributed phases, determined from the spectra of the fresh catalyst after calcination, reduction and oxidation^a^

Sample	IS (mm s^−1^)	QS (mm s^−1^)	*Γ* (HWHM) (mm s^−1^)	Fe species	Area (%)	Attributed phase
Fresh catalyst	0.42(1)	0.34(1)	0.17	Fe^3+^	100	FePO_4_-tdm
Reduced with CH_4_	0.37(1)	1.00(2)	0.16	Fe^3+^	25(2)	Fe_7_(PO_4_)_6_
	0.35(8)	0.67(8)	0.15	Fe^3+^	17(2)	FePO_4_-low quartz
	1.28(7)	2.53(8)	0.17	Fe^2+^	27(4)	Fe_2_P_2_O_7_
	1.29(1)	2.29(4)	0.17	Fe^2+^	19(1)	
	1.25(3)	1.35(7)	0.17	Fe^2+^	3(1)	
Oxidized with O_2_	0.41(6)	1.01(6)	0.23	Fe^3+^	28(4)	α-Fe_3_(P_2_O_7_)_2_
	0.40(1)	0.60(3)	0.22	Fe^3+^	46(4)	
	1.02(8)	2.23(9)	0.21	Fe^2+^	6(1)	Fe_2_P_2_O_7_
	1.15(4)	2.71(9)	0.22	Fe^2+^	7(1)	
	0.31(1)	0.24(5)	0.20	Fe^3+^	13(2)	FePO_4_-tdm
Oxidized with N_2_O	0.31(1)	0.78(1)	0.20	Fe^3+^	34(3)	α-Fe_3_(P_2_O_7_)_2_
	0.51(2)	0.81(2)	0.23	Fe^3+^	29(3)	
	1.15(2)	2.00(5)	0.20	Fe^2+^	6(1)	Fe_2_P_2_O_7_
	1.08(2)	2.74(6)	0.20	Fe^2+^	7(1)	
	0.30(1)	0.22(3)	0.16	Fe^3+^	24(1)	FePO_4_-tdm
Oxidized with H_2_O	0.39(1)	1.03(1)	0.20	Fe^3+^	33(1)	α-Fe_3_(P_2_O_7_)_2_
	0.39(1)	0.56(1)	0.23	Fe^3+^	56(2)	
	1.14(2)	2.75(5)	0.22	Fe^2+^	6(1)	Fe_2_P_2_O_7_
	1.07(3)	2.16(6)	0.20	Fe^2+^	5(1)	

a The isomer shifts are expressed relative to α-Fe at room temperature.

The phase parameters of the fresh catalyst confirm that only the FePO_4_-tdm phase is present in the catalyst, in agreement with literature and previous work.^23,24,37^ Two Fe^2+^ species which are observed after the reduction of the fresh catalyst under methane at 500 °C, are attributable to the Fe_2_P_2_O_7_ phase.^22^ A 17% contribution from a FePO_4_ low quartz phase is evident, most likely the result of the unreduced phase present in the catalyst.^37^ The effect of oxygen atmosphere on the phase formation is reflected by the Mössbauer spectrum for the O_2_ oxidized catalyst (Fig. 8), which shows a 74% spectral area due to a Fe^3+^ iron phase and a weaker Fe^2+^ component with IS of 1.15 mm s^−1^ and QS of 2.71 mm s^−1^. These values are in accordance with the values of Fe^3+^ and Fe^2+^ components in the α-phase of the catalyst, α-Fe_3_(P_2_O_7_)_2_. The XRD profile of the catalyst also supports this assignment. In addition, a trydimite like FePO_4_ phase is observed with a spectral area of 13%.

**Fig. 9:**
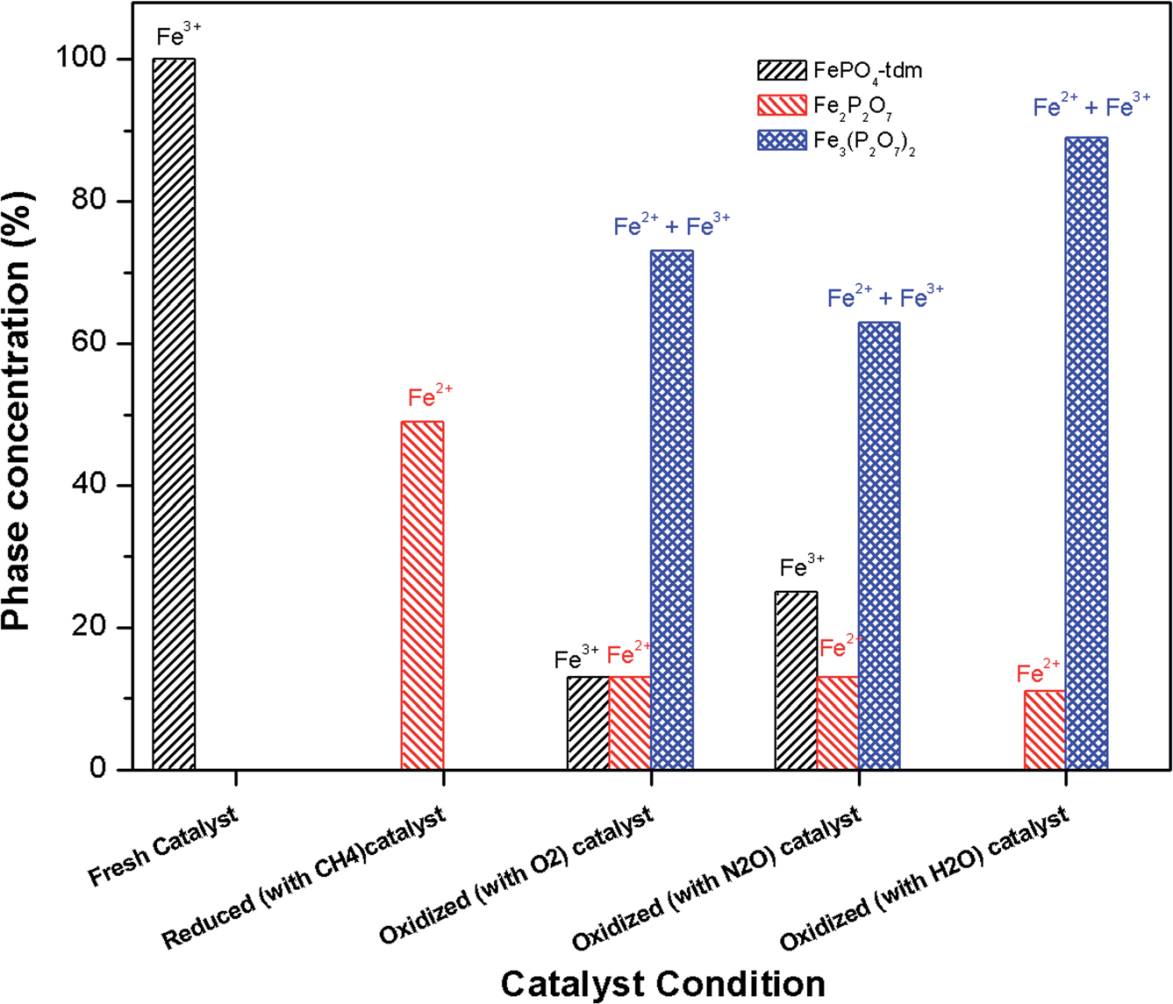
Phase quantification of fresh, reduced (with CH_4_) and oxidized (with N_2_O, O_2_ and H_2_O) FePO_4_ catalyst determined from the Mössbauer spectra shown in Fig. 8.

**Fig. 10:**
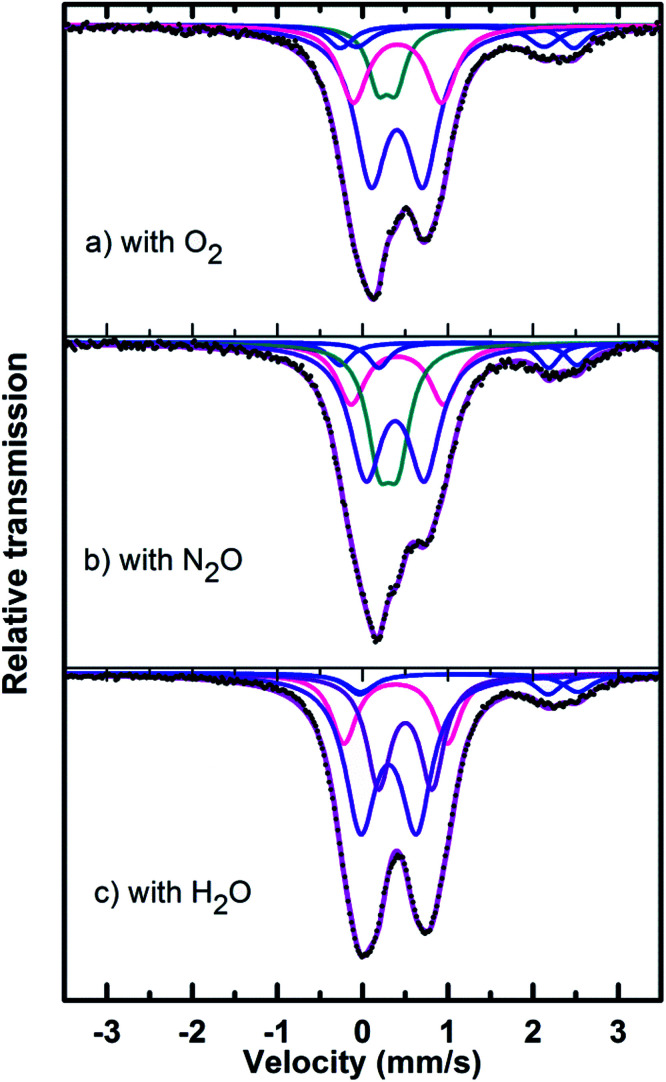
Mössbauer spectra of used catalysts after reaction with (a) O_2_, (b) N_2_O and (c) H_2_O.

The Mössbauer spectrum obtained for the oxidized catalyst with N_2_O (Fig. 8), showed a 12% contribution from components with IS and QS values characteristic of Fe^2+^ (Fig. 9 and Table 2) which can be assigned to the Fe_2_P_2_O_7_ phase. In addition, the spectrum showed a 18% Fe^3+^ component with parameters corresponding to the α-phase together with a mixture of 44% Fe^2+^ in the Fe_2_P_2_O_7_ phase. A Fe^3+^ species, with IS and QS values which were not characteristic of a typical FePO_4_-tdm phase in the oxidized catalyst, with a 26% site fraction is observed.^22,37^ The Mössbauer spectrum of the catalyst oxidized with H_2_O, showed Fe^2+^ components with a combined 11% intensity, which can be assigned to the Fe_2_P_2_O_7_ phase. The spectrum also shows the presence of the Fe^3+^ species with a 88% site fraction (Fig. 10). The respective IS and QS values of this ferric species are in agreement with the Fe^3+^ species in the various iron phosphate type phases, namely the α-phase.^20,22,23^

In the Powder XRD diffractogram of the used catalyst after the reaction with O_2_ atmosphere, two major peaks are evident at 34.2° and 35.1° which can be attributed to the Fe_2_P_2_O_7_ phase, but this region also coincides with an overlap of the most intense peak of the α-phase.^20,37^ In the Mössbauer data of the used catalysts (Fig. 10), the α-Fe_3_(P_2_O_7_)_2_ phase, with a Fe^3+^ spectral component, was the dominant phase (74%). The appearance of the Fe_2_P_2_O_7_ phase was also observed, with ferric and ferrous species each contributing towards an 11% total site fraction.^43^ The formation of the α-phase results from the transformation between the FePO_4_-tdm and Fe_2_P_2_O_7_ phases, which takes place reversibly, depending on strength of the redox atmosphere^20,23^ and the fact that the α-Fe_3_(P_2_O_7_)_2_ phase is a mixed ferric and ferrous pyrophosphate consisting of both Fe_4_(P_2_O_7_)_3_ and Fe_2_P_2_O_7_.^37,43^

The diffractogram obtained for the used catalyst after the reaction with N_2_O (ESI, Fig. S5†), showed an intense, sharp peak at 2*θ* = 34.5°. The Mössbauer data (Fig. 10) for the used catalyst after the reaction with N_2_O showed typical of Fe^3+^ species (88%) and a Fe^2+^ species (12%) with parameters which correlate with the FePO_4_-low quartz and Fe_7_(PO_4_)_6_ phases. In addition, there is a component (22%) with IS and QS values corresponding to Fe^2+^ which we identified as Fe_2_P_2_O_7_ phase. These results showed an enhancement towards the α-phase after reaction with N_2_O atmosphere. The absence of the β-phase during the oxidation reactions has been observed in previous studies, especially during the oxidative dehydrogenation of isobutyric acid with a water co-feed over FePO_4_ catalyst.^22,23,43^ In literature, it has been reported that an FePO_4_ catalyst consisting of the quartz type phase as the precursor, undergoes phase transformation during a catalytic reaction involving the oxidative dehydrogenation of isobutyric acid to form Fe_7_(PO_4_)_6_ and the α-phase (Table 3).^18,23^

**Table 3:** Mössbauer parameters, isomer shift (IS), electric quadrupole splitting (QS), line width *Γ* (HWHM) and the attributed phases of the spent catalyst after oxidation in O_2_, N_2_O and H_2_O atmospheres^a^

Oxidizing atmosphere	IS (mm s^−1^)	QS (mm s^−1^)	*Γ* (HWHM) (mm s^−1^)	Fe species	Area(%)	Attributed phase
O_2_	0.40(1)	0.61(2)	0.23	Fe^3+^	51(2)	α-Fe_3_(P_2_O_7_)_2_
	0.41(2)	1.04(2)	0.20	Fe^2+^	23(2)	
	1.03(8)	2.20(5)	0.25	Fe^2+^	7(1)	Fe_2_P_2_O_7_
	1.11(4)	2.74(4)	0.18	Fe^2+^	5(1)	
	0.29(1)	0.21(2)	0.14	Fe^3+^	12(2)	FePO_4_-tdm
N_2_O	0.38(1)	0.68(3)	0.23	Fe^3+^	44(3)	Fe_7_(PO_4_)_6_
	0.40(2)	1.09(3)	0.20	Fe^3+^	18(3)	FePO_4_-low quartz
	1.19(4)	2.00(5)	0.15	Fe^2+^	6(1)	Fe_2_P_2_O_7_
	1.13(2)	2.78(4)	0.16	Fe^2+^	6(1)	
	0.30(1)	0.22(8)	0.17	Fe^3+^	26(1)	FePO_4_-tdm
H_2_O	0.30(1)	0.65(5)	0.22	Fe^3+^	45(2)	α-Fe_3_(P_2_O_7_)_2_
	0.50(1)	0.63(1)	0.17	Fe^3+^	26(1)	α-Fe_3_(P_2_O_7_)_2_
	0.39(1)	1.20(2)	0.18	Fe^2+^	17(2)	
	1.14(2)	2.75(5)	0.22	Fe^2+^	6(1)	Fe_2_P_2_O_7_
	1.25(3)	2.54(8)	0.20	Fe^2+^	5(1)	

a The isomer shifts are expressed relative to α-Fe at room temperature.

**Fig. 11:**
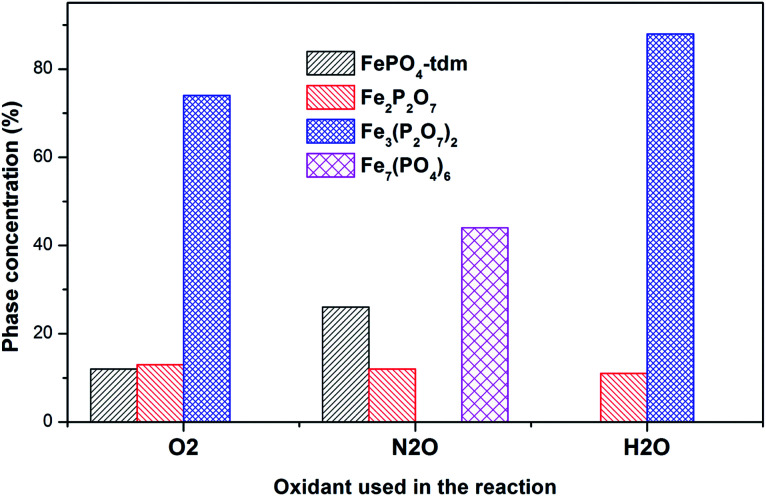
Phase quantification of used catalysts after reaction with O_2_, N_2_O and H_2_O, determined from the Mössbauer spectra.

The diffractogram of the used catalyst using H_2_O as an oxidant showed the presence of Fe_2_P_2_O_7_ phase along with α-phase. The Mössbauer spectrum, after using H_2_O as an oxidant (Fig. 10), showed Fe^2+^ and Fe^3+^ components with a relative intensities of 11% and 71% (Fig. 11), which can be assigned to the α-Fe_3_(P_2_O_7_)_2_ phase.^20,22,23^ Similar to the catalyst obtained after reaction under N_2_O atmosphere, no evidence of β-phase was observed after the reaction with H_2_O also.^17,18,24^ It has been reported that formation of the β-phase is dependent on the catalyst structure and redox atmosphere.^43,44^ Obviously, water plays a role in avoiding high reduction atmospheres. Similar observation was reported in literature,^22,37^ that the addition of the water co-feed in the reaction, blocks the formation of the less selective β-phase. In summary, the XRD and Mössbauer spectra of the fresh and used catalysts oxidized in the presence of N_2_O and H_2_O showed that the α-phase was formed in high quantity^22^ when the formation of β-phase was suppressed.

Panov *et al.*^3,12^ assessed the methane partial oxidation by nitrous oxide in the processes in which the α-oxygen sites were created at 160 °C temperature over FeZSM-5 zeolite. They discovered that the reactions occur through a hydrogen abstraction mechanism, making methoxy or hydroxy groups bounded to the α-sites. When the same reaction is performed with heating to 160 °C, they verified that at CH_4_ : N_2_O molar ratio equal to 1 : 1, the reactions directly provide methanol. Wood *et al.* studied the mechanism of “catalytic” oxidation reactions of methane by nitrous oxide over Fe-ZSM-5 zeolite, and concluded that the primary products of methane oxidation are methoxy groups bounded to active iron (*i.e.*, Fe–OCH_3_). As can be seen, the Fe^2+^ cations of α-sites are activated by nitrous oxide generating adsorbed oxidant species (*i.e.*, Fe^3+^–O˙−)_α_-sites, which convert methane to adsorbed methanol. Afterwards, adsorbed methanol can be converted to dimethyl ether and water. Beznis *et al.*^45^ assessed the activity of Co–ZSM-5 solid catalysts on reactions of partial oxidation of methane. They found that methanol production proportionally increased in relation to surface area of catalyst, which can be increased treating the catalyst with NaOH. These authors discovered that the active sites (*i.e.*, cobalt oxidic species, such as Co_3_O_4_ and CoO, present on catalyst external surface) were also proportionally formed in relation to surface area of solid catalyst. In the presence of N_2_O, CH_4_ would be oxidized by peroxo species MO_2_ formed by the reaction of N_2_O with a Fe=O centre. Fe=O centres may be the active sites for methanol formation.^3,17,46^ The kinetic results obtained on the oxidation of CH_4_ by N_2_O showed that carbon oxides are probably primary products and may also stem from methanol or formaldehyde as secondary products. The direct pathway to carbon oxides could be ascribed to the existence of surface sites where the intermediate oxygenates expected are strongly attached and not allowed to desorb in the gas phase. In contrast, formation of methanol or formaldehyde would rather correspond to oxygenate precursors more easily desorbed because less retained at the catalyst surface.

In literature, it is well established that Fe based catalysts entail different chemical properties considering those of their other individual monometallic components, *e.g.* achieving the synergistic effects in methane activation reactions. In general, ZSM-5 catalysts show a higher activity for methane activation than other supports. Panov and co-workers^3,4^ showed the methane oxidation over Fe-ZSM-5 catalysts by N_2_O at 200 °C and showed that methanol that formed *via* methane oxidation by α-oxygen, CH_4_ + (Fe^III^–O˙^−^)α, migrated from α-sites, initiating new reaction cycles. At 200 °C, a 4 h run provided a turn over frequency (TOF) of 5.5 × 10^−3^ μmol_MeOH_ g_cat_ h^−1^. Beznis and co-workers^45,47^ showed that the selective activation of methane towards methanol over Co–ZSM-5 can be influenced by altering the micro-and meso-porosity of the zeolite material. They showed a linear relationship between the ZSM-5 surface area and the amount of methanol produced (5.5 × 10^−3^ μmol_MeOH_ g_cat_ h^−1^) over Co–ZSM-5 from methane and oxygen at 250 °C (Table 4).

**Table 4:** Comparison of catalytic performance of Fe based catalysts for methane activation reaction reported in literature

S. no	Catalyst	Oxidant	Temperature (°C)	Methanol TOF (μmol_MeOH_ g_cat_ h^−1^)	Reference
1	2% Fe–ZSM5	O_2_	300	6.3 × 10^−4^	4
2	2% Fe–ZSM5^a^	N_2_O	200	5.5 × 10^−3^	3
3	2% Fe–ZSM5	N_2_O	550	7.1 × 10^−3^	19
4	0.5% Fe–SIL-1	N_2_O	550	8 × 10^−3^	2
5	FePO_4_/MCM-41	O_2_	400	7.5 × 10^−4^	14
6	FePO_4_/SBA-15	O_2_	500	1.2 × 10^−4^	48
7	2% Co–ZSM5	O_2_	250	1.5 × 10^−4^	45 and 47
8	0.5% Fe–SiO_2_	O_2_	500	2.1 × 10^−4^	49 and 50
9	FePO_4_-tdm	O_2_	300	5.3 × 10^−3^	This work
10	FePO_4_-tdm	N_2_O	300	12.3 × 10^−3^	This work

a Batch reactor, under a pressure of 2 bar.

Zhang and co-workers^48–50^ showed that the iron species introduced into mesoporous silica SBA-15 could catalyze the selective oxidation of CH_4_ to methanol by O_2_ and that the catalyst with a Fe content of 0.5 wt% provided the highest single-pass yield (2.1 × 10^−4^ μmol_MeOH_ g_cat_ h^−1^). The TOF towards methanol formation decreased with increasing Fe content. They also studied SBA-15-supported iron phosphate (FePO_4_) for the partial oxidation of CH_4_ with O_2_. The SBA-15-supported FePO_4_ catalysts exhibit higher CH_4_ conversion and MeOH selectivity than the unsupported and the MCM-41-supported ones in the partial oxidation of CH_4_ with O_2_.^14,48,50^ The catalyst with a loading amount of 5 wt% shows the highest MeOH selectivity at a given CH_4_ conversion and the highest MeOH formation rate based on the amount of FePO_4_ in the catalyst. It is likely that the improved catalytic performances of the SBA-15-supported samples are related to the enhanced redox properties of FePO_4_ species, the large porous diameter and the high inertness of SBA-15.^48^ With compared to the other catalysts reported in literature, the FePO_4_-tdm phase catalysts showed in this work exhibited a high activity towards methanol *i.e.*, 12.3 × 10^−3^ μmol_MeOH_ g_cat_ h^−1^ using N_2_O as an oxidant. This catalyst also showed a high activity with O_2_ as an oxidant (5.3 × 10^−3^ μmol_MeOH_ g_cat_ h^−1^).

## 4 Conclusion

A high yield of methanol was observed over FePO_4_ when using N_2_O as oxidant. The methane conversion with O_2_ and N_2_O increased steeply with temperature but increased much slower when H_2_O was applied as an oxidizing agent. Furthermore, the maximum CH_4_ consumption (17 mol%) was obtained when oxygen had been used as the oxidant at 500 °C, the highest in this study. The selectivity towards methanol was very good at lower methane conversions (elevated flow rates). Additionally, when a comparison was made at all the levels of CH_4_ consumption, it was the highest when N_2_O was used as an oxidant. The present results clearly show that the selective oxidation of methane over FePO_4_ is influenced by the nature of the oxidizing agent. Furthermore, the fresh catalyst possessed a rough crystalline morphology with particle sizes ranging from 50 to 80 nm, and with a homogeneous dispersion of crystallites. After reduction, agglomeration of these nanoparticles was observed. After the oxidation with O_2_, the crystalline nature was retained, but with separate agglomerated bulk particles present. From TPRO profiles, it was evident that the type of oxidant, used in re-oxidation, influenced the pathway of oxidation for a reduced FePO_4_ catalyst.

Mössbauer spectroscopy, complemented with powder X-ray diffraction, proved to be a very sensitive tool in providing an understanding of the phase transformations of FePO_4_ material using O_2_, H_2_O and N_2_O as oxidizing agents for the selective conversion of methane to methanol. The Mössbauer data provided evidence that the Fe_2_P_2_O_7_ phase was dominant in the reduced catalyst sample, while its amount decreased five-fold after the oxidation with O_2_ due to the formation of α-domains. In the Mössbauer data of the used catalysts, the α-Fe_3_(P_2_O_7_)_2_ phase, with a Fe^3+^ spectral component, was the dominant phase (74%). The appearance of the Fe_2_P_2_O_7_ phase was also observed, with ferric and ferrous species each contributing towards an 11% total site fraction. The formation of the α-phase results from the transformation between the FePO_4_-tdm and Fe_2_P_2_O_7_ phases, which takes place reversibly, depending on strength of the redox atmosphere and the fact that the α-Fe_3_(P_2_O_7_)_2_ phase is a mixed ferric and ferrous pyrophosphate consisting of both Fe_4_(P_2_O_7_)_3_ and Fe_2_P_2_O_7_. In summary, the XRD and Mössbauer spectra of the fresh and used catalysts oxidized in the presence of N_2_O and H_2_O showed that the α-phase was formed in high quantity when the formation of β-phase was suppressed. When compared to the other catalysts reported in literature, the FePO_4_-tdm phase catalysts showed in this work exhibited a high activity towards methanol *i.e.*, 12.3 × 10^−3^ μmol_MeOH_ g_cat_ h^−1^ using N_2_O as an oxidant. This catalyst also showed a high activity with O_2_ as an oxidant (5.3 × 10^−3^ μmol_MeOH_ g_cat_ h^−1^).

## Conflicts of interest

There are no conflicts to declare.

## Supplementary Material

RA-009-C9RA02327E-s001
