# Supplementary material for: The effect of oxidant species on direct, non-syngas conversion of methane to methanol over an FePO4 catalyst material
Source: RSC Adv. 2019 Sep 30;9(53):30989–1003. doi: 10.1039/c9ra02327e (PMC9072299; doi:10.1039/c9ra02327e)
Supplement: RA-009-C9RA02327E-s001 [file RA-009-C9RA02327E-s001.pdf]

Electronic Supplementary Material (ESI) for RSC Advances.
This journal is © The Royal Society of Chemistry 2019

Electronic Supplementary Information

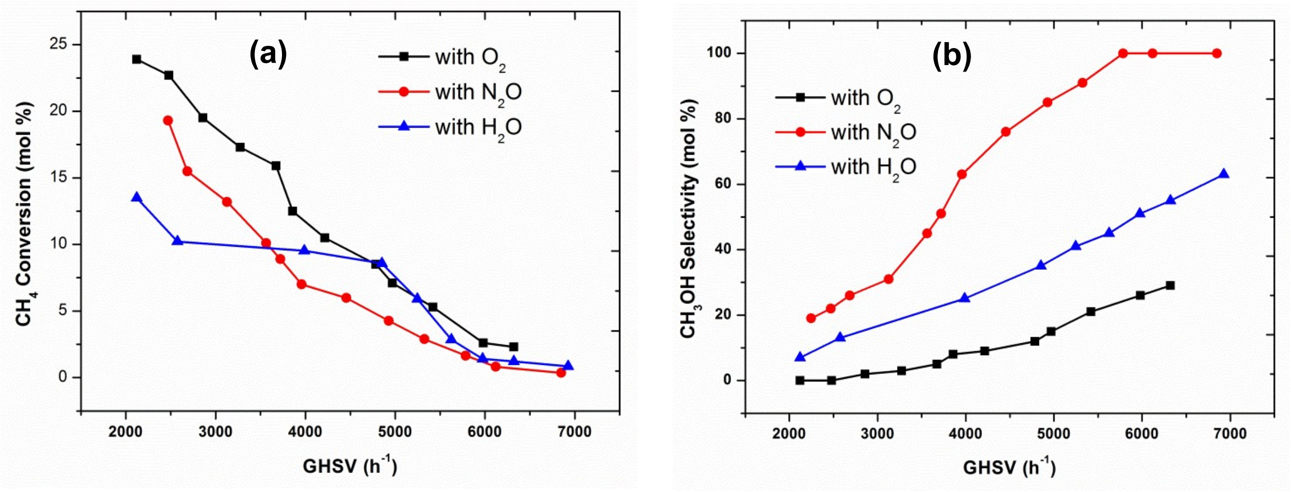

Figure S1: Influence of flow rate on (a) methane conversion and (b) methanol selectivity in partial oxidation of methane over $FePO_4$ catalyst (GHSV = 2000-7000 h^{-1}, temperature = 300 °C and at methane to oxidant ratio of 1:1)

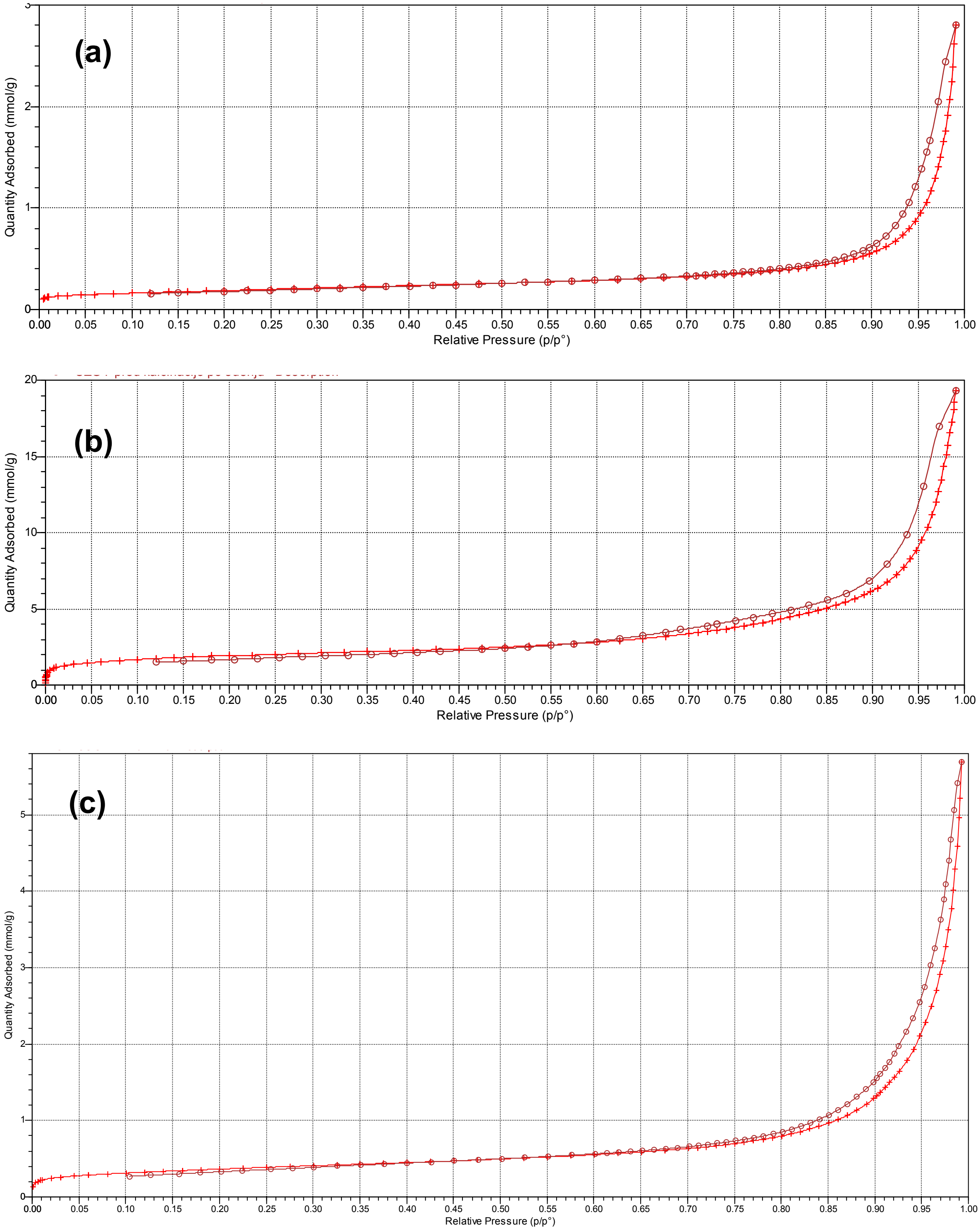
(a)
Quantity Adsorbed (mmol/g)
Relative Pressure (p/p°)
0
1
2
0.00
0.05
0.10
0.15
0.20
0.25
0.30
0.35
0.40
0.45
0.50
0.55
0.60
0.65
0.70
0.75
0.80
0.85
0.90
0.95
1.00
(b)
Quantity Adsorbed (mmol/g)
Relative Pressure (p/p°)
0
5
10
15
20
(c)
Quantity Adsorbed (mmol/g)
Relative Pressure (p/p°)
0
1
2
3
4
5

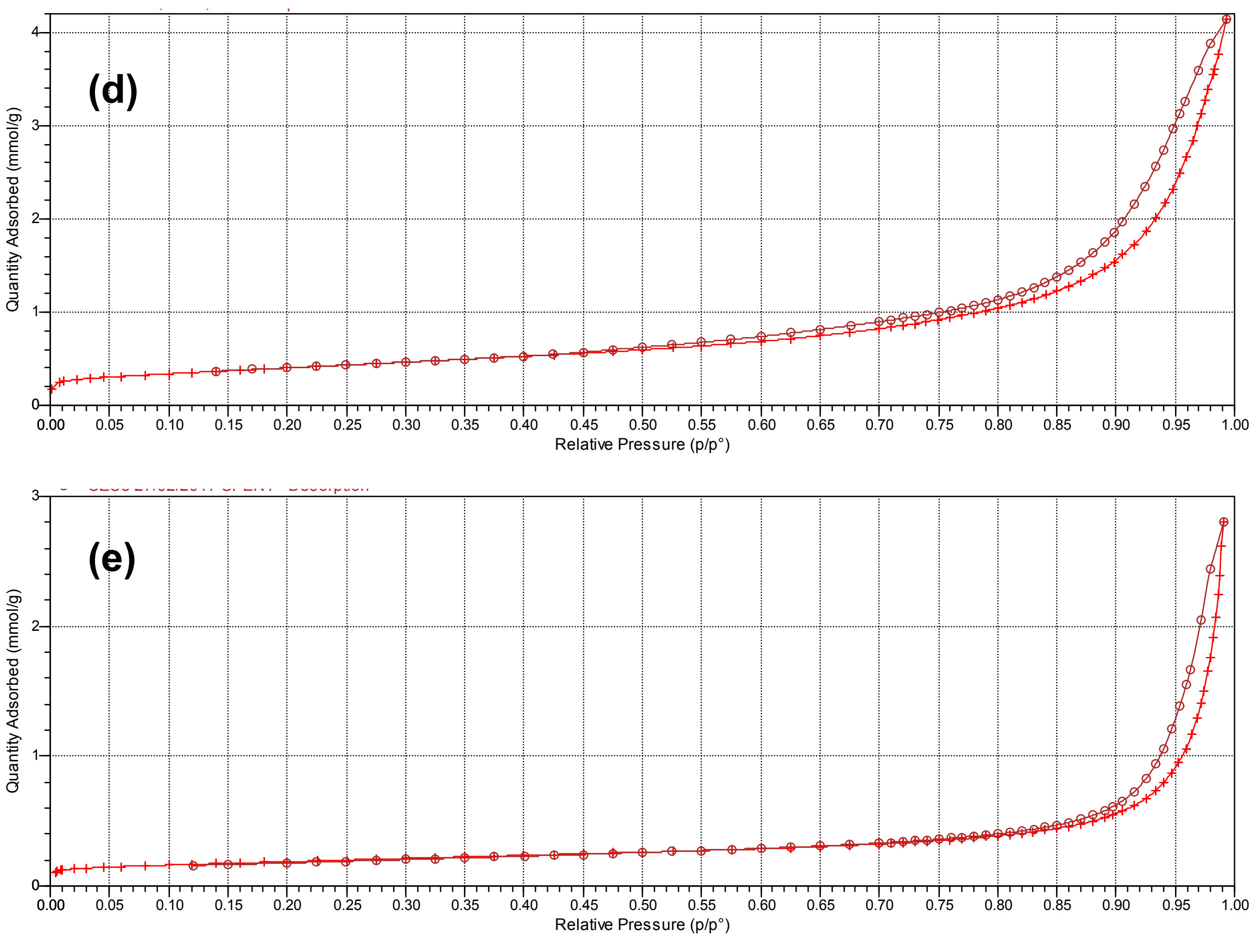

Figure S2: N_2 physisorption isotherms of $FePO_4$ catalyst. (a) fresh, (b) reduced (with CH_4) and oxidised with (c) N_2O, (d) O_2 and (e) H_2O.

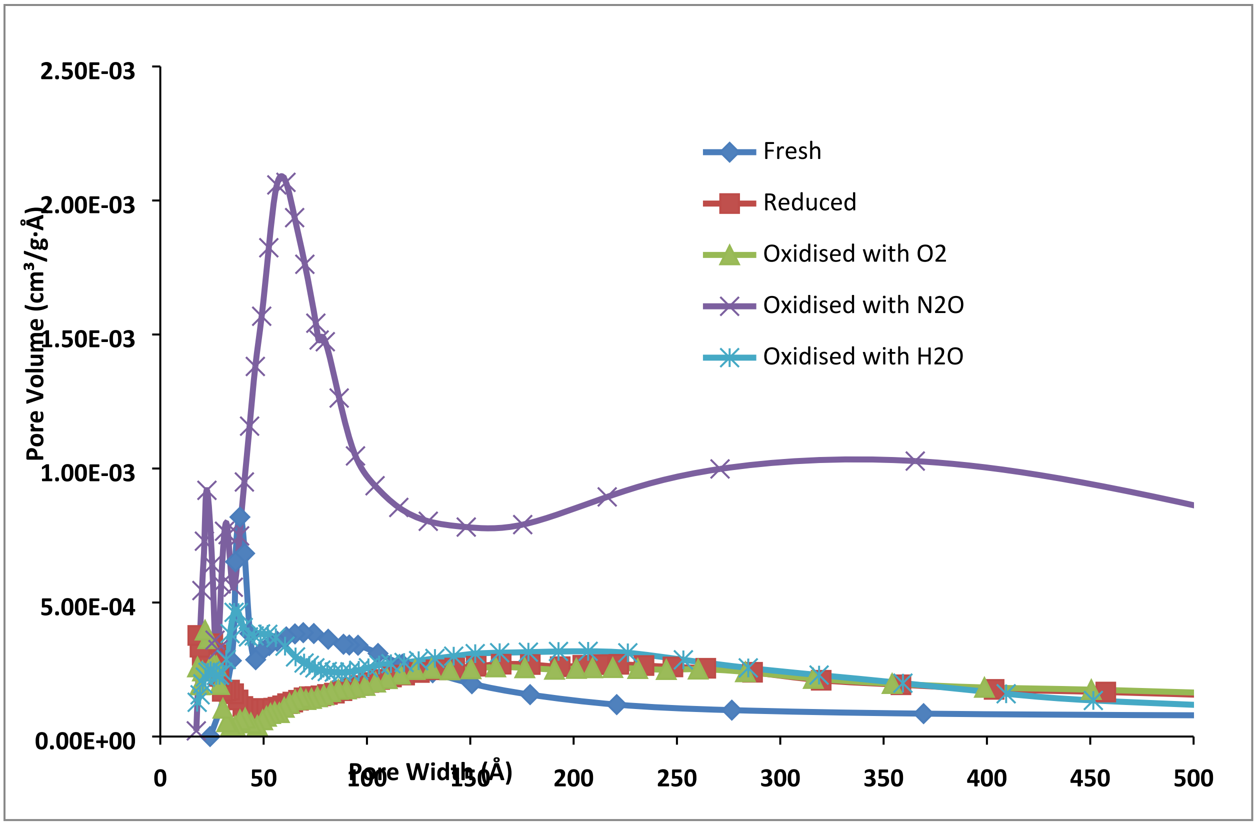

Figure S3: Pore size distribution plot of $FePO_4$ catalyst

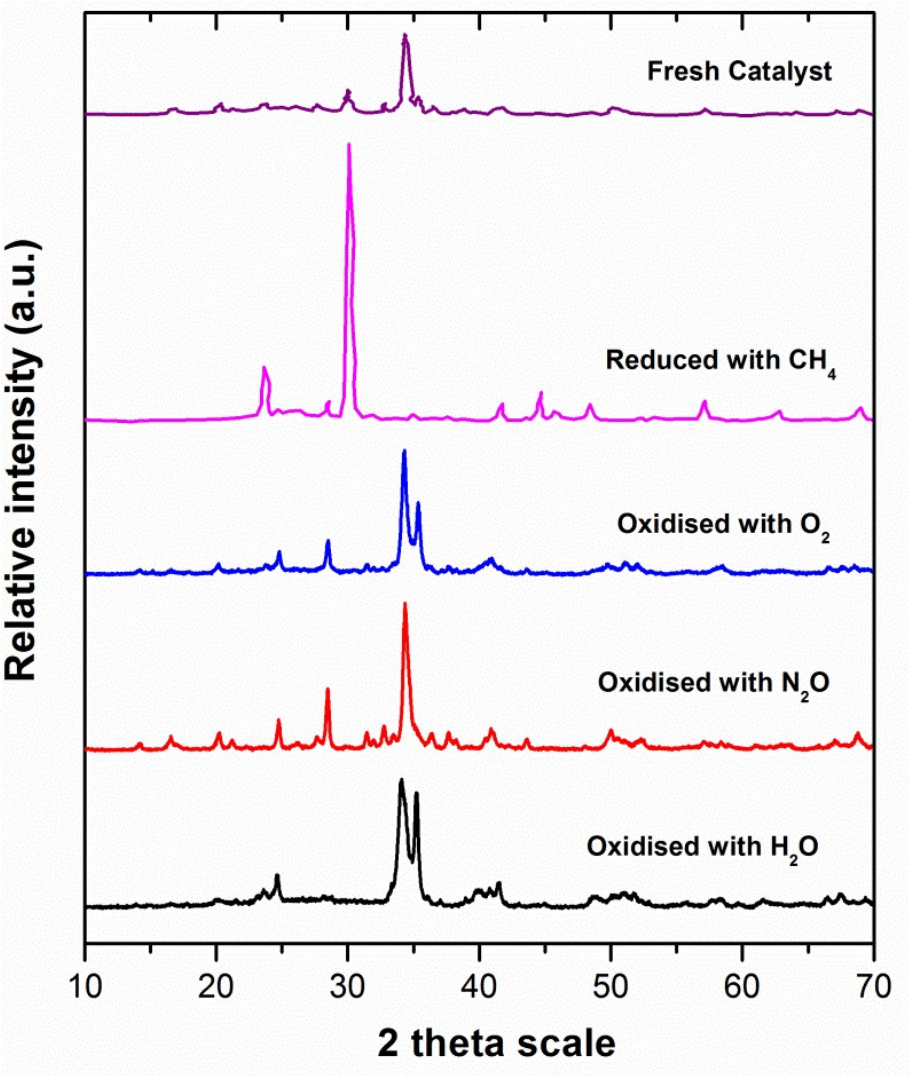

Figure S4: Powder X-ray diffraction of $FePO_4$ catalyst under various treatments [37] (Reprinted with permission from Springer Link)

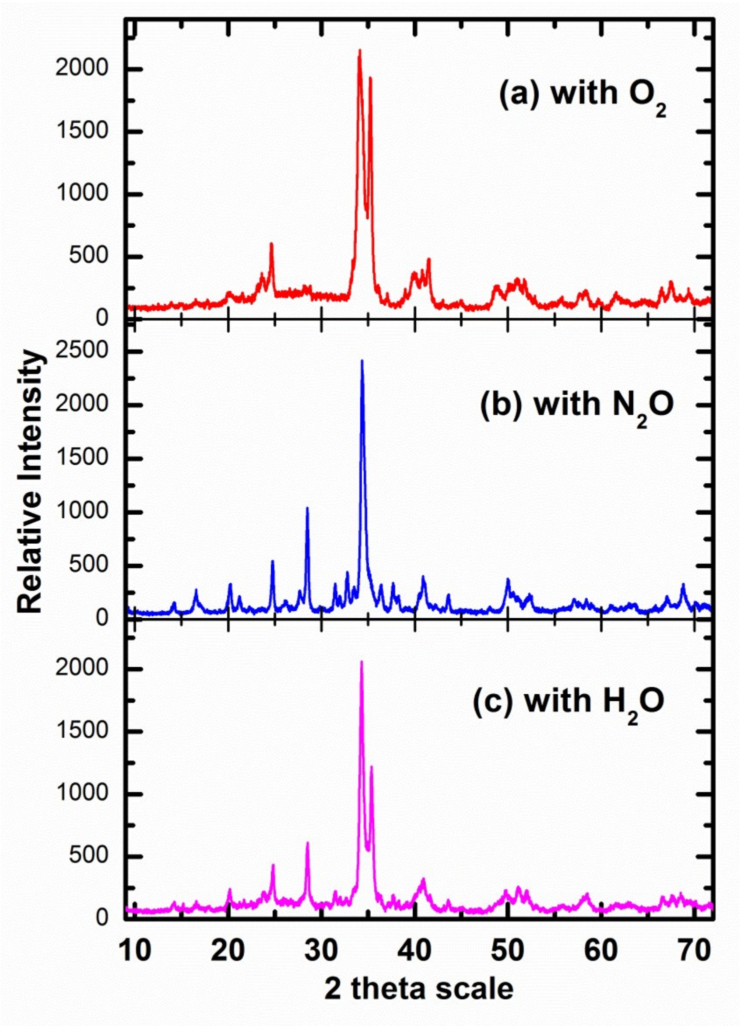

Figure S5 : Powder X-ray diffraction patterns of the used catalysts after reaction with (a) O_2, (b) N_2O and (c) H_2O.[37] (Reprinted with permission from Springer Link)
